# Fuzzy random sensitivity analysis for the overall structure reliability of reinforced concrete freezing wellbores in deep alluvium based on hidden Markov model

**DOI:** 10.1038/s41598-024-65914-4

**Published:** 2024-07-06

**Authors:** Yafeng Yao, Yan Zhu, Yongheng Li, Wei Wang, Zhemei Zhang

**Affiliations:** 1 School of Construction Engineering, Nantong Vocational University, Nantong, 226001 China; 2 AI and BIM Integrated Intelligent Construction Engineering Technology Research and Development Center, Nantong Vocational University, Nantong, 226007 China; 3 Anhui Key Laboratory of Building Structure and Underground Engineering, Anhui Jianzhu University, Hefei, 210037 China; 4 Department of Architecture and Civil Engineering, City University of Hong Kong, Kowloon, 999077 Hong Kong China

**Keywords:** Sensitivity analysis, Fuzzy random reliability, Overall structure, Big data, Hidden Markov model mode, Applied mathematics, Computer science, Civil engineering

## Abstract

To address the shortcomings of traditional reliability theory in characterizing the stability of deep underground structures, the advanced first order second moment of reliability was improved to obtain fuzzy random reliability, which is more consistent with the working conditions. The traditional sensitivity analysis model was optimized using fuzzy random optimization, and an analytical calculation model of the mean and standard deviation of the fuzzy random reliability sensitivity was established. A big data hidden Markov model and expectation-maximization algorithm were used to improve the digital characteristics of fuzzy random variables. The fuzzy random sensitivity optimization model was used to confirm the effect of concrete compressive strength, thick-diameter ratio, reinforcement ratio, uncertainty coefficient of calculation model, and soil depth on the overall structural reliability of a reinforced concrete double-layer wellbore in deep alluvial soil. Through numerical calculations, these characteristics were observed to be the main influencing factors. Furthermore, while the soil depth was negatively correlated, the other influencing factors were all positively correlated with the overall reliability. This study provides an effective reference for the safe construction of deep underground structures in the future.

## Introduction

With the gradual exploitation of global coal resources, shallow resources have been almost exhausted, so a large number of projects are turning to deep strata for excavation. In specific deep mining processes, accidents involving frozen shafts have increased, and their reliability has become a research focus^1,2^. In contrast to the shallow shafts used previously, the practice of deep alluvium reinforced-concrete frozen shaft engineering is more complicated because of the external environment. This results in obvious fuzzy randomness of the external shaft load and ultimate resistance and the overall reliability of the frozen shaft shows high uncertainty. However, traditional reliability calculations generally adopt a load-resistance model, and the results obtained only represent the final safety and reliability degree of the structure, thus failing to clarify the influence of different parameters on the degree of reliability^3,4^. Therefore, it is necessary to conduct a sensitivity analysis of the overall structural reliability. Based on the reliability calculation, the degree of influence of different parameters on structural reliability was analyzed to distinguish the main and secondary factors that guide construction practice.

In recent studies the sensitivity analysis of structural reliability has been explored. Babazadeh et al.^5^ transformed the nonlinear limit state function into a linear limit state function by Monte Carlo simulation. On this basis, random variables were introduced, and the reliability sensitivity parameters of the mouth brooder algorithm were adopted. Wang et al.^6^ proposed an improved extended polynomial chaotic expansion method. This method models and simultaneously propagates both random and cognitive random variables, constructing a composite mapping from each cognitive variable to the response PDF. The global sensitivity index of the PDF was derived with respect to the distributed parameters. Tian et al.^7^ adopted a subset simulation method to develop a Bayesian updated structural reliability method, and established an efficient reliability-monitoring sensitivity analysis framework. Xiao et al.^8^ proposed a new effective sampling method to estimate the fault conditions and reliability sensitivity indices using a two-stage Markov chain Monte Carlo simulation. Considering the influence of random factors, Li et al.^9^ adopted Monte Carlo and adaptive Krieger-Monte Carlo simulation methods to analyze the reliability of cold-formed curtain wall glass. Based on this, a reliability sensitivity analysis of the curtain wall glass was conducted to evaluate the influence of random changes in different parameters on the reliability of the curtain wall. Proppe et al.^10^ proposed a method that extended the moving particle method to a sensitivity analysis based on local reliability. It was based completely on the evaluated samples for reliability estimation and avoided the repeated evaluation of performance functions.

In summary, most previous studies have built sensitivity analysis models using the basic theory of reliability analysis, ignoring the inherent uncertainty of complex environments and structural parameters. The result has been inaccurate sensitivity acquisition and deviation in guiding practical projects. Other studies have used probabilistic statistical theory to analyze the structural reliability and sensitivity; however, this only considers the randomness of the engineering and does not consider the fuzziness of the structure. In addition, in the sensitivity analysis, the sample space data are small and the calculation is complicated. As a result engineering applications are not extensive.

Therefore, based on the big data algorithm environment, this study regards the freezing and shaft walls of deep underground frozen wellbores as fuzzy random force fields. It first establishes a big data fuzzy random reliability model of the entire structure of the reinforced concrete frozen wellbore. Finally, it conducts a fuzzy random sensitivity analysis of the reliability of the entire structure of the wellbore during the construction period. It is important to guide the rationality of the shaft design and improve the safety and reliability of deep underground engineering.

## Theory

### HMM model

The main goal of big data analysis is to obtain unknown and potential information patterns and rules through a large number of effective information means and calculation methods to extract the depth value of the data and provide effective data for industrial decision-making and engineering practice^11–14^.

The HMM is a doubly random process. It is not possible to observe its state directly, which can be implicitly derived from the observed vector^15,16^.

The main components of the HMM are as follows.

1. State of the model
  Let the set of states be $s=\left\{{s}_{1},{s}_{2},\cdots ,{s}_{N}\right\}$ and the state at time *t* be ${q}_{t}\in S$. These states can then be transferred to each other.
2. State transition matrix
  $A=({a}_{ij}{)}_{N\times N}$ is a state matrix that describes the manner in which transitions occur between states. *a*_*ij*_ is the probability of state transition.
3. Observed model values
  The observed values are set for $V=\left\{{v}_{1},{v}_{2},\cdots ,{v}_{M}\right\}$. When the state transition at time *t* is complete, the model generates an observable output ${y}_{t}\in V$.
4. The probability distribution matrix is output.
  $B=({b}_{ij}{)}_{N\times M}$ is a probability distribution function matrix describing the output.
  In this matrix, ${b}_{ij}={b}_{i}(j)={b}_{i}({v}_{j})=P({y}_{t}={v}_{j}\left|{q}_{t}={s}_{i}\right.)$ is the probability that the output is *v*_*j*_ when the state is *s*_*i*_ at time *t*.
5. Initial state distribution
  Let $\pi =\left\{{\pi }_{1},{\pi }_{2},\cdots ,{\pi }_{N}\right\}$ be the initial state distribution of the model, where ${\pi }_{i}=P({q}_{1}={s}_{i})$. Therefore, a complete HMM can be used to represent all the parameters using $\lambda =\left\{A,B,\pi \right\}$.

### Optimization of big data reliability algorithms

The traditional reliable AFOSM uses a Taylor series to expand its equation of state, with the first moment representing the mean value and the second moment representing the variance. Therefore, the reliability index of the overall structure of the approximation algorithm is the quotient of the two, as expressed in Eq. (1).

1 $$\beta \approx \frac{{\mu }_{Z}}{{\sigma }_{Z}}=\frac{g({\mu }_{{x}_{1}},{\mu }_{{x}_{2}},\cdot \cdot \cdot ,{\mu }_{{x}_{m}})}{\sqrt{{{\sum }_{i=1}^{m}(\frac{\partial g}{\partial {x}_{i}})}^{2}{{\sigma }^{2}}_{{x}_{i}}}}$$

**Figure 1:**
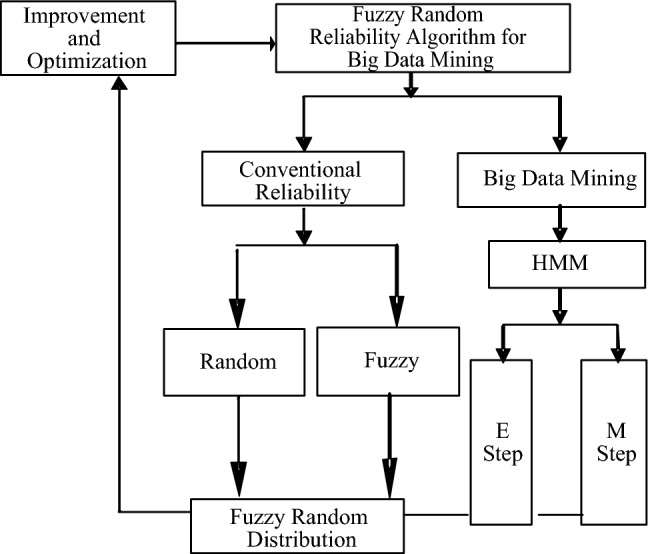
Reliability optimization with big data algorithm.

Therefore, based on the HMM of big data, an EM algorithm was adopted to improve the traditional reliability calculation method. The steps of the EM algorithm are as follows:

1. Expectation step: calculate the maximum likelihood estimate based on the initial values of the parameters or model parameters of the last iteration of ${\theta }^{(n)}$, as shown in Eq. (2).
  2 $${Q}_{i}({z}^{(i)}):=p({z}^{(i)}\left|{x}^{(i)};\theta \right.)$$
2. Maximization step: fix *Q*(*z*), When the likelihood of the data is at its maximum, the parameter expectation estimation is calculated, as shown in Eq. (3).
  3 $$\theta : = argmax\mathop \sum \limits_{i} \mathop \sum \limits_{z\left( i \right)} Q\left( i \right)\left( {z^{\left( i \right)} } \right)\log \frac{{p\left( {x^{\left( i \right)} ,z^{\left( i \right)} ;\theta } \right)}}{{Q_{i} \left( {z^{\left( i \right)} } \right)}}$$
3. The above steps are repeated until convergence. The big-data EM algorithm flowchart is shown in Fig. 1.

### Fuzzy random reliability of frozen wellbore structure

In China, the freezing section of a deep alluvial frozen shaft is typically double-walled. According to a large amount of engineering practice data, the inner and outer walls of a frozen shaft during construction bear mainly freezing and hydrostatic pressures, respectively. During the operation period, grouting reinforcement is usually performed between the inner and outer shaft walls, and the double shaft can be regarded as a whole. Owing to the friction caused by permafrost thaw settling and soil drainage settlement in the later stages, the permanent ground pressure is generally considered as the design control load in the safety check calculation of the entire shaft.

Therefore, the overall force on the inner and outer walls of the deep frozen shaft can be regarded as a fuzzy random force field, and the static earth pressure of the deep alluvial layer and the overall resistance of the reinforced concrete double wall can be analyzed using fuzzy random analysis to obtain fuzzy random equations, as shown in Eqs. (4) and (5)^17,18^.

4 $$\tilde{P} = 0.013\tilde{H}$$

where $\widetilde{P}$ is the static earth pressure suffered by the entire wellbore during operation, and $\widetilde{H}$ is the soil depth of different well sections. Considering the uncertainty of the parameters of the deep alluvium, they are fuzzy random variables.

5 $$\tilde{R}_{k} = \tilde{\Theta }_{R} \cdot (m_{N} \tilde{\lambda }_{N} \tilde{R}_{aN} + m_{W} \tilde{\lambda }_{W} \tilde{R}_{aW} + \tilde{\mu }_{N} R_{gN} + \tilde{\mu }_{W} R_{gW} )$$

where $\widetilde{{R}_{k}}$ is the fuzzy random value of the ultimate bearing capacity of the reinforced concrete double wall; ${\widetilde{\Theta }}_{R}$ is the uncertainty coefficient of the calculation model; ${\widetilde{R}}_{aN} and {\widetilde{R}}_{aW}$ are the compressive strength of the inner and outer shaft wall concrete axes, respectively; ${\widetilde{\mu }}_{N} and {\widetilde{\mu }}_{W}$ are the ratio of the annular reinforcements of the inner and outer shaft walls, respectively. ${\widetilde{\lambda }}_{N} and {\widetilde{\lambda }}_{W}$ are the thickness-to-diameter ratio of the inner and outer shafts, respectively. All of these are fuzzy random variables. ${m}_{N} and {m}_{W}$ are the inner and outer wall concrete strength enhancement coefficients, respectively; ${R}_{gN} and {R}_{gW}$ are the strengths of the steel bars in the inner and outer shaft walls, respectively; considering the degree of influence on the ultimate bearing capacity, the above values are expressed in fixed form.

Based on the above big-data EM algorithm, the load and resistance equations are introduced into the reliability equation of fuzzy randomization, and the fuzzy random function of the overall structure of a deep alluvial reinforced concrete double-layer wellbore is established using the HMM model, as shown in Eq. (6).

6 $$\tilde{Z} = \tilde{R} - \tilde{S} = \theta :[\tilde{\Theta }_{R} \cdot (m_{N} \tilde{\lambda }_{N} \tilde{R}_{aN} + m_{W} \tilde{\lambda }_{W} \tilde{R}_{aW} + \tilde{\mu }_{N} R_{gN} + \tilde{\mu }_{W} R_{gW} ) - 0.013\tilde{H}]$$

In Eq. (6), the corresponding parameters have the same meaning as before.

Big data from engineering examples show that the triangular fuzzy factor can be used to characterize the uncertainty of the fuzzy random threshold $\widetilde{b}$, which is closer to the actual working conditions. Therefore, the fuzzy random state equation for the overall structure can be expressed as follows:

7 $$\tilde{\Theta }_{R} \cdot (m_{N} \tilde{\lambda }_{N} \tilde{R}_{aN} + m_{W} \tilde{\lambda }_{W} \tilde{R}_{aW} + \tilde{\mu }_{N} R_{gN} + \tilde{\mu }_{W} R_{gW} ) - 0.013\tilde{H} = \tilde{b}$$

Similarly, the corresponding fuzzy stochastic equations for the effective and failure states of the overall structure are expressed as

8 $$\tilde{\Theta }_{R} \cdot (m_{N} \tilde{\lambda }_{N} \tilde{R}_{aN} + m_{W} \tilde{\lambda }_{W} \tilde{R}_{aW} + \tilde{\mu }_{N} R_{gN} + \tilde{\mu }_{W} R_{gW} ) - 0.013\tilde{H} > \tilde{b}$$

9 $$\tilde{\Theta }_{R} \cdot (m_{N} \tilde{\lambda }_{N} \tilde{R}_{aN} + m_{W} \tilde{\lambda }_{W} \tilde{R}_{aW} + \tilde{\mu }_{N} R_{gN} + \tilde{\mu }_{W} R_{gW} ) - 0.013\tilde{H} < \tilde{b}$$

According to the big data algorithm, for the fuzzy random equation of state (Eq. 7) of the overall structure, the cut set region of the constraint level *α* is considered, and its interval equation is expressed as:

10 $$\tilde{R}_{\alpha } - \tilde{S}_{\alpha } = \theta :\left[ {\tilde{\Theta }_{R} \cdot (m_{N} \tilde{\lambda }_{N} \tilde{R}_{aN} + m_{W} \tilde{\lambda }_{W} \tilde{R}_{aW} + \tilde{\mu }_{N} R_{gN} + \tilde{\mu }_{W} R_{gW} ) - 0.013\tilde{H}} \right]_{\alpha } = \tilde{b}_{\alpha }$$

where, $$\tilde{S}_{\alpha } = \left\{ {\left( {0.013\tilde{H}} \right)_{\alpha }^{ - } ,\left( {0.013\tilde{H}} \right)_{\alpha }^{ + } } \right\},$$

$$\begin{gathered} \tilde{R}_{\alpha } = \{ \theta :[\tilde{\Theta }_{R} \cdot (m_{N} \tilde{\lambda }_{N} \tilde{R}_{aN} + m_{W} \tilde{\lambda }_{W} \tilde{R}_{aW} + \tilde{\mu }_{N} R_{gN} + \tilde{\mu }_{W} R_{gW} )]_{\alpha }^{ - } ,\, \hfill \\ \theta :[\tilde{\Theta }_{R} \cdot (m_{N} \tilde{\lambda }_{N} \tilde{R}_{aN} + m_{W} \tilde{\lambda }_{W} \tilde{R}_{aW} + \tilde{\mu }_{N} R_{gN} + \tilde{\mu }_{W} R_{gW} )]_{\alpha }^{ + } \} , \hfill \\ \end{gathered}$$

$$\tilde{b}_{\alpha } = [\tilde{b}^{ - } ,\;\tilde{b}^{ + } ]_{\alpha } = \left[ {b + (\alpha - 1)d,\;b + (1 - \alpha )d} \right].$$

According to the fuzzy interval algorithm, the fuzzy interval of the decomposed function is

11 $$\tilde{Z}_{\alpha }^{ - } = \tilde{R}_{\alpha }^{ - } - \tilde{S}_{\alpha }^{ + } = \theta :\left\{ {\left[ {\tilde{\Theta }_{R} \cdot (m_{N} \tilde{\lambda }_{N} \tilde{R}_{aN} + m_{W} \tilde{\lambda }_{W} \tilde{R}_{aW} + \tilde{\mu }_{N} R_{gN} + \tilde{\mu }_{W} R_{gW} )} \right]_{\alpha }^{ - } - \left[ {0.013\tilde{H}} \right]_{\alpha }^{ + } } \right\} = \tilde{b}_{\alpha }^{ - }$$

12 $$\tilde{Z}_{\alpha }^{ + } = \tilde{R}_{\alpha }^{ + } - \tilde{S}_{\alpha }^{ - } = \theta :\left\{ {\left[ {\tilde{\Theta }_{R} \cdot (m_{N} \tilde{\lambda }_{N} \tilde{R}_{aN} + m_{W} \tilde{\lambda }_{W} \tilde{R}_{aW} + \tilde{\mu }_{N} R_{gN} + \tilde{\mu }_{W} R_{gW} )} \right]_{\alpha }^{ + } - \left[ {0.013\tilde{H}} \right]_{\alpha }^{ - } } \right\} = \tilde{b}_{\alpha }^{{^{ + } }}$$

According to the algorithm flow of the HMM of big data, the fuzzy random function of the overall well wall is expanded into a Taylor series and substituted into the partial derivative of the corresponding variable^19,20^, and the following results can then be obtained:

13 $$\begin{aligned} \tilde{Z} \approx & g(u_{{x_{1} }} ,\mu_{{x_{2} }} , \ldots ,\mu_{{x_{n} }} ) + \sum\limits_{i = 1}^{n} {(x_{i} - \mu_{{x_{i} }} )\frac{\partial g}{{\partial x_{i} }}} \\ = & g\;(u_{{\tilde{H}}} ,\mu_{{\tilde{R}_{aN} }} ,\mu_{{\tilde{R}_{aW} }} ,\mu_{{\tilde{\mu }_{N} }} ,\mu_{{\tilde{\mu }_{W} }} ,\mu_{{\tilde{\lambda }_{N} }} ,\mu_{{\tilde{\lambda }_{W} }} , - 0.013(H - \mu_{H} ) \\ & + \tilde{\Theta }_{{\tilde{R}}} (R_{aN} - \mu_{{\tilde{R}_{aN} }} )(m_{N} \tilde{\lambda }_{N} ) + \tilde{\Theta }_{{\tilde{R}}} (R_{aW} - \mu_{{\tilde{R}_{aW} }} )(m_{W} \tilde{\lambda }_{W} ) + \tilde{\Theta }_{R} (\mu_{N} - \mu_{{\tilde{\mu }_{N} }} )(R_{gN} ) \\ & + \tilde{\Theta }_{R} (\mu_{W} - \mu_{{\tilde{\mu }_{W} }} )(R_{gw} ) + \tilde{\Theta }_{R} (\lambda_{N} - \mu_{{\tilde{\lambda }_{N} }} )(m_{N} \tilde{R}_{aN} ) + \tilde{\Theta }_{R} (\lambda_{W} - \mu_{{\tilde{\lambda }_{W} }} )(m_{W} \tilde{R}_{aW} ) \\ \end{aligned}$$

The fuzzy random reliability index for big-data analysis is expressed as follows:

14 $$\begin{gathered} \tilde{\beta } = \frac{{\tilde{\mu }_{Z} }}{{\tilde{\sigma }_{Z} }} \approx \theta :\{ [\tilde{\Theta }_{R} \cdot (m_{N} \mu_{{\tilde{\lambda }_{N} }} \mu_{{\tilde{R}_{aN} }} + m_{W} \mu_{{\tilde{\lambda }_{W} }} \mu_{{\tilde{R}_{aW} }} + \mu_{{\tilde{\mu }_{N} }} R_{gN} + \mu_{{\tilde{\mu }_{W} }} R_{gW} ) \hfill \\ - 0.013\mu_{{\tilde{H}}} ]/\left. {[(\frac{{\partial \tilde{Z}}}{{\partial \tilde{H}}}} \right|_{{\tilde{\mu }_{H} }} \cdot \tilde{\sigma }_{H} )^{2} + (\left. {\frac{{\partial \tilde{Z}}}{{\partial \tilde{R}_{aN} }}} \right|_{{\tilde{\mu }_{{\tilde{R}_{aN} }} }} \cdot \tilde{\sigma }_{{\tilde{R}_{aN} }} )^{2} + (\left. {\frac{{\partial \tilde{Z}}}{{\partial \tilde{R}_{aW} }}} \right|_{{\tilde{\mu }_{{\tilde{R}_{aW} }} }} \cdot \tilde{\sigma }_{{\tilde{R}_{aW} }} )^{2} + \hfill \\ (\left. {\frac{{\partial \tilde{Z}}}{{\partial \tilde{\mu }_{N} }}} \right|_{{\tilde{\mu }{}_{{\tilde{\mu }_{N} }}}} \cdot \tilde{\sigma }_{{\tilde{\mu }_{N} }} )^{2} + (\left. {\frac{{\partial \tilde{Z}}}{{\partial \tilde{\mu }_{W} }}} \right|_{{\tilde{\mu }_{{\tilde{\mu }_{W} }} }} \cdot \tilde{\sigma }_{{\tilde{\mu }_{W} }} )^{2} + (\left. {\frac{{\partial \tilde{Z}}}{{\partial \tilde{\lambda }_{N} }}} \right|_{{\tilde{\mu }_{{\tilde{\lambda }_{N} }} }} \cdot \tilde{\sigma }_{{\tilde{\lambda }_{N} }} )^{2} + (\left. {\frac{{\partial \tilde{Z}}}{{\partial \tilde{\lambda }_{W} }}} \right|_{{\tilde{\mu }_{{\tilde{\lambda }_{W} }} }} \cdot \tilde{\sigma }_{{\tilde{\lambda }_{W} }} )^{2} + (\left. {\frac{{\partial \tilde{Z}}}{{\partial \tilde{\lambda }_{W} }}} \right|_{{\tilde{\mu }_{{\tilde{\lambda }_{W} }} }} \cdot \tilde{\sigma }_{{\tilde{\lambda }_{W} }} )^{2} ]^{\frac{1}{2}} \} \hfill \\ \end{gathered}$$

In order to better illustrate the advantages of fuzzy random reliability, the freezing double-layer shaft lining in deep alluvial in Lianghuai mining is taken as an example. The traditional reliability and fuzzy random reliability of the overall structure are shown in Table 1^21,22^.

**Table 1:** Fuzzy random reliability compared with conventional reliability.

Depth (m)	Conventional reliability index	Conventional reliability	Fuzzy random reliability index		Fuzzy random reliability	
			$\beta^{ - }$	$\beta^{ + }$	$P_{s}^{ - }$	$P_{s}^{ + }$
391–437	2.9683	0.9985	1.9300	2.9677	0.9732	0.9985
437–502	3.2401	0.9994	2.1083	3.2388	0.9825	0.9994
502–530	3.1528	0.9991	2.0198	3.1213	0.9783	0.9991
530–564	2.9905	0.9986	1.9899	2.9888	0.9767	0.9986

### Fuzzy stochastic reliability sensitivity optimization model

#### Fuzzy random reliability sensitivity

Structural reliability sensitivity reflects the importance of the basic parameters for the safety of the entire structure. Usually, the importance of each parameter is determined by calculating the partial derivative of the structural parameters and failure probability^23,24^.

However, traditional reliability sensitivity is primarily based on the basic theory of traditional reliability calculations, ignoring the inherent uncertainty of complex environments and structural parameters and not considering the fuzziness and randomness of deep underground engineering.

To meet the need for fuzzy random reliability combined with actual working conditions, fuzzy random optimization of the sensitivity should also be conducted. Fuzzy random sensitivity is mainly concerned with the importance of these uncertain parameters in engineering, affecting the overall structural safety and reliability^25,26^. For this reason, the fuzzy random reliability sensitivity can be expressed by Eq. (15).

15 $$\left( {\frac{{\partial \tilde{P}_{f} }}{{\partial \tilde{\mu }_{{x_{i} }} }},\frac{{\partial \tilde{P}_{f} }}{{\partial \tilde{\sigma }_{{x_{i} }} }}} \right)$$

When the characteristic distribution of the parameters is biased, the fuzzy random reliability sensitivity can be transformed into^27,28^:

16 $$\left( {\frac{{\partial \tilde{P}_{f} }}{{\partial \tilde{\beta }}} \cdot \frac{{\partial \tilde{\beta }}}{{\partial \tilde{\mu }_{{x_{i} }} }},\frac{{\partial \tilde{P}_{f} }}{{\partial \tilde{\beta }}} \cdot \frac{{\partial \tilde{\beta }}}{{\partial \tilde{\sigma }_{{x_{i} }} }}} \right)$$

Combined with the fuzzy random reliability theory, the fuzzy random failure probability and fuzzy random reliability index are substituted into Eq. (16), and the following can be obtained:

17 $$\frac{{\partial \tilde{P}_{f} }}{{\partial \tilde{\beta }}}\; \cdot \frac{{\partial \tilde{\beta }}}{{\partial \tilde{\mu }_{{x_{i} }} }} \approx \frac{{\partial \left( {1 - \frac{1}{{\sqrt {2\pi } }}\int_{ - \infty }^{\beta } {e^{{ - \frac{1}{2}x^{2} }} } dx} \right)}}{{\partial \tilde{\beta }}} \cdot \frac{{\partial \left( {\frac{{\tilde{\mu }_{Z} }}{{\tilde{\sigma }_{Z} }}} \right)}}{{\partial \tilde{\mu }_{{x_{i} }} }} = - \frac{{\left( {\frac{{\partial \tilde{Z}}}{{\partial \tilde{x}_{i} }}} \right)}}{{\sqrt {2\pi } \tilde{\sigma }_{Z} }}\exp \left[ { - \frac{1}{2}\left( {\frac{{\tilde{\mu }_{Z} }}{{\tilde{\sigma }_{Z} }}} \right)^{2} } \right]$$

18 $$\frac{{\partial \tilde{P}_{f} }}{{\partial \tilde{\beta }}}\; \cdot \frac{{\partial \tilde{\beta }}}{{\partial \tilde{\sigma }_{{x_{i} }} }} \approx \frac{{\partial \left( {1 - \frac{1}{{\sqrt {2\pi } }}\int_{ - \infty }^{\beta } {e^{{ - \frac{1}{2}x^{2} }} } dx} \right)}}{{\partial \tilde{\beta }}} \cdot \frac{{\partial \left( {\frac{{\tilde{\mu }_{Z} }}{{\tilde{\sigma }_{Z} }}} \right)}}{{\partial \tilde{\sigma }_{{x_{i} }} }} = - \frac{{\left( {\frac{{\partial \tilde{Z}}}{{\partial \tilde{x}_{i} }}} \right)^{2} \tilde{\mu }_{Z} \tilde{\sigma }_{{x_{i} }} }}{{\sqrt {2\pi } \tilde{\sigma }_{Z}^{3} }}\exp \left[ { - \frac{1}{2}\left( {\frac{{\tilde{\mu }_{Z} }}{{\tilde{\sigma }_{Z} }}} \right)^{2} } \right]$$

Taking the horizontal cut set of Eqs. (17) and (18), whose constraint level is, the fuzzy random reliability sensitivity model can be expressed as

19 $$\left( {\frac{{\partial \tilde{P}_{f} }}{{\partial \tilde{\mu }_{{x_{i} }} }}} \right)_{\alpha } = \bigcup\limits_{\alpha \in (0,1]} {\alpha \left[ {\mathop {\left( {\frac{{\partial \tilde{P}_{f} }}{{\partial \tilde{\mu }_{{x_{i} }} }}} \right)}\nolimits_{\alpha }^{ - } ,\mathop {\left( {\frac{{\partial \tilde{P}_{f} }}{{\partial \tilde{\mu }_{{x_{i} }} }}} \right)}\nolimits_{\alpha }^{ + } } \right]}$$

20 $$\left( {\frac{{\partial \tilde{P}_{f} }}{{\partial \tilde{\sigma }_{{x_{i} }} }}} \right)_{\alpha } = \bigcup\limits_{\alpha \in (0,1]} {\alpha \left[ {\mathop {\left( {\frac{{\partial \tilde{P}_{f} }}{{\partial \tilde{\sigma }_{{x_{i} }} }}} \right)}\nolimits_{\alpha }^{ - } ,\mathop {\left( {\frac{{\partial \tilde{P}_{f} }}{{\partial \tilde{\sigma }_{{x_{i} }} }}} \right)}\nolimits_{\alpha }^{ + } } \right]}$$

Among them:

21 $$\mathop {\left( {\frac{{\partial \tilde{P}_{f} }}{{\partial \tilde{\mu }_{{x_{i} }} }}} \right)}\nolimits_{\alpha }^{ - } = \inf \left\{ {\bigcup\limits_{\alpha \in (0,1]} \alpha \left[ { - \frac{{\left( {\frac{{\partial \tilde{Z}}}{{\partial \tilde{x}_{i} }}} \right)}}{{\sqrt {2\pi } \tilde{\sigma }_{Z} }}\exp \left[ { - \frac{1}{2}\left( {\frac{{\tilde{\mu }_{Z} }}{{\tilde{\sigma }_{Z} }}} \right)^{2} } \right]} \right]_{\alpha } } \right\}$$

22 $$\mathop {\left( {\frac{{\partial \tilde{P}_{f} }}{{\partial \tilde{\mu }_{{x_{i} }} }}} \right)}\nolimits_{\alpha }^{ + } = \sup \left\{ {\bigcup\limits_{\alpha \in (0,1]} \alpha \left[ { - \frac{{\left( {\frac{{\partial \tilde{Z}}}{{\partial \tilde{x}_{i} }}} \right)}}{{\sqrt {2\pi } \tilde{\sigma }_{Z} }}\exp \left[ { - \frac{1}{2}\left( {\frac{{\tilde{\mu }_{Z} }}{{\tilde{\sigma }_{Z} }}} \right)^{2} } \right]} \right]_{\alpha } } \right\}$$

23 $$\mathop {\left( {\frac{{\partial \tilde{P}_{f} }}{{\partial \tilde{\sigma }_{{x_{i} }} }}} \right)}\nolimits_{\alpha }^{ - } = \inf \left\{ {\bigcup\limits_{\alpha \in (0,1]} \alpha \left[ { - \frac{{\left( {\frac{{\partial \tilde{Z}}}{{\partial \tilde{x}_{i} }}} \right)^{2} \tilde{\mu }_{Z} \tilde{\sigma }_{{x_{i} }} }}{{\sqrt {2\pi } \tilde{\sigma }_{Z}^{3} }}\exp \left[ { - \frac{1}{2}\left( {\frac{{\tilde{\mu }_{Z} }}{{\tilde{\sigma }_{Z} }}} \right)^{2} } \right]} \right]_{\alpha } } \right\}$$

24 $$\mathop {\left( {\frac{{\partial \tilde{P}_{f} }}{{\partial \tilde{\sigma }_{{x_{i} }} }}} \right)}\nolimits_{\alpha }^{ + } = \sup \left\{ {\bigcup\limits_{\alpha \in (0,1]} \alpha \left[ { - \frac{{\left( {\frac{{\partial \tilde{Z}}}{{\partial \tilde{x}_{i} }}} \right)^{2} \tilde{\mu }_{Z} \tilde{\sigma }_{{x_{i} }} }}{{\sqrt {2\pi } \tilde{\sigma }_{Z}^{3} }}\exp \left[ { - \frac{1}{2}\left( {\frac{{\tilde{\mu }_{Z} }}{{\tilde{\sigma }_{Z} }}} \right)^{2} } \right]} \right]_{\alpha } } \right\}$$

In the above equation, $\mathit{inf}(\cdot )$ and $\mathit{sup}(\cdot )$ are the minimum and maximum values, respectively, of the fuzzy random horizontal cut set interval.

#### Fuzzy random reliability sensitivity model of frozen wellbore mold overall structure

Using the HMM and EM algorithms for big data, the partial derivatives of each main fuzzy random variable were obtained according to the fuzzy random function of the overall structure of the reinforced concrete double-layer wellbore with a deep alluvial layer. The relevant digital feature function was substituted into Eqs. (19)–(24). The main parameters for a fuzzy random sensitivity optimization model of the reliability of a reinforced concrete double-layer shaft structure with a deep alluvial layer can then be obtained^29,30^.

1. Fuzzy random sensitivity optimization model for compressive strength of concrete axis.
  25 $$\mathop {\left( {\frac{{\partial \tilde{P}_{f} }}{{\partial \tilde{\mu }_{{R_{{\text{a}}} }} }}} \right)}\nolimits_{\alpha }^{ - } = \inf \left\{ {\bigcup\limits_{\alpha \in (0,1]} \alpha \left[ { - \frac{{\theta :[\tilde{\Theta }_{R} \cdot (m_{N} \tilde{\lambda }_{N} + m_{W} \tilde{\lambda }_{W} )]}}{{\sqrt {2\pi } \tilde{\sigma }_{Z} }}\exp \left[ { - \frac{1}{2}\left( {\frac{{\tilde{\mu }_{Z} }}{{\tilde{\sigma }_{Z} }}} \right)^{2} } \right]} \right]_{\alpha } } \right\}$$
  26 $$\mathop {\left( {\frac{{\partial \tilde{P}_{f} }}{{\partial \tilde{\mu }_{{R_{{\text{a}}} }} }}} \right)}\nolimits_{\alpha }^{ + } = \sup \left\{ {\bigcup\limits_{\alpha \in (0,1]} \alpha \left[ { - \frac{{\theta :[\tilde{\Theta }_{R} \cdot (m_{N} \tilde{\lambda }_{N} + m_{W} \tilde{\lambda }_{W} )]}}{{\sqrt {2\pi } \tilde{\sigma }_{Z} }}\exp \left[ { - \frac{1}{2}\left( {\frac{{\tilde{\mu }_{Z} }}{{\tilde{\sigma }_{Z} }}} \right)^{2} } \right]} \right]_{\alpha } } \right\}$$
  27 $$\mathop {\left( {\frac{{\partial \tilde{P}_{f} }}{{\partial \tilde{\sigma }_{{R_{a} }} }}} \right)}\nolimits_{\alpha }^{ - } = \inf \left\{ {\bigcup\limits_{\alpha \in (0,1]} \alpha \left[ { - \frac{{\theta :[\tilde{\Theta }_{R} \cdot (m_{N} \tilde{\lambda }_{N} + m_{W} \tilde{\lambda }_{W} )]^{2} \tilde{\mu }_{Z} \tilde{\sigma }_{H} }}{{\sqrt {2\pi } \tilde{\sigma }_{Z}^{3} }}\exp \left[ { - \frac{1}{2}\left( {\frac{{\tilde{\mu }_{Z} }}{{\tilde{\sigma }_{Z} }}} \right)^{2} } \right]} \right]_{\alpha } } \right\}$$
  28 $$\mathop {\left( {\frac{{\partial \tilde{P}_{f} }}{{\partial \tilde{\sigma }_{{R_{a} }} }}} \right)}\nolimits_{\alpha }^{ + } = \sup \left\{ {\bigcup\limits_{\alpha \in (0,1]} \alpha \left[ { - \frac{{\theta :[\tilde{\Theta }_{R} \cdot (m_{N} \tilde{\lambda }_{N} + m_{W} \tilde{\lambda }_{W} )]^{2} \tilde{\mu }_{Z} \tilde{\sigma }_{H} }}{{\sqrt {2\pi } \tilde{\sigma }_{Z}^{3} }}\exp \left[ { - \frac{1}{2}\left( {\frac{{\tilde{\mu }_{Z} }}{{\tilde{\sigma }_{Z} }}} \right)^{2} } \right]} \right]_{\alpha } } \right\}$$
2. Fuzzy random sensitivity model for circumferential reinforcement ratio of shaft wall.
  29 $$\mathop {\left( {\frac{{\partial \tilde{P}_{f} }}{{\partial \tilde{\mu }_{\mu } }}} \right)}\nolimits_{\alpha }^{ - } = \inf \left\{ {\bigcup\limits_{\alpha \in (0,1]} \alpha \left[ {{ - }\frac{{\theta :[\tilde{\Theta }_{R} \cdot (R_{gN} + R_{gW} )]}}{{\sqrt {2\pi } \tilde{\sigma }_{Z} }}\exp \left[ { - \frac{1}{2}\left( {\frac{{\tilde{\mu }_{Z} }}{{\tilde{\sigma }_{Z} }}} \right)^{2} } \right]} \right]_{\alpha } } \right\}$$
  30 $$\mathop {\left( {\frac{{\partial \tilde{P}_{f} }}{{\partial \tilde{\mu }_{\mu } }}} \right)}\nolimits_{\alpha }^{ + } = \sup \left\{ {\bigcup\limits_{\alpha \in (0,1]} \alpha \left[ { - \frac{{\theta :[\tilde{\Theta }_{R} \cdot (R_{gN} + R_{gW} )]}}{{\sqrt {2\pi } \tilde{\sigma }_{Z} }}\exp \left[ { - \frac{1}{2}\left( {\frac{{\tilde{\mu }_{Z} }}{{\tilde{\sigma }_{Z} }}} \right)^{2} } \right]} \right]_{\alpha } } \right\}$$
  31 $$\mathop {\left( {\frac{{\partial \tilde{P}_{f} }}{{\partial \tilde{\sigma }_{\mu } }}} \right)}\nolimits_{\alpha }^{ - } = \inf \left\{ {\bigcup\limits_{\alpha \in (0,1]} \alpha \left[ { - \frac{{\theta :[\tilde{\Theta }_{R} \cdot (R_{gN} + R_{gW} )]^{2} \tilde{\mu }_{Z} \tilde{\sigma }_{\theta } }}{{\sqrt {2\pi } \tilde{\sigma }_{Z}^{3} }}\exp \left[ { - \frac{1}{2}\left( {\frac{{\tilde{\mu }_{Z} }}{{\tilde{\sigma }_{Z} }}} \right)^{2} } \right]} \right]_{\alpha } } \right\}$$
  32 $$\mathop {\left( {\frac{{\partial \tilde{P}_{f} }}{{\partial \tilde{\sigma }_{\mu } }}} \right)}\nolimits_{\alpha }^{ + } = \sup \left\{ {\bigcup\limits_{\alpha \in (0,1]} \alpha \left[ { - \frac{{\theta :[\tilde{\Theta }_{R} \cdot (R_{gN} + R_{gW} )]^{2} \tilde{\mu }_{Z} \tilde{\sigma }_{\theta } }}{{\sqrt {2\pi } \tilde{\sigma }_{Z}^{3} }}\exp \left[ { - \frac{1}{2}\left( {\frac{{\tilde{\mu }_{Z} }}{{\tilde{\sigma }_{Z} }}} \right)^{2} } \right]} \right]_{\alpha } } \right\}$$
3. Fuzzy random sensitivity model for the thickness-to-diameter ratio of the wellbore.
  33 $$\mathop {\left( {\frac{{\partial \tilde{P}_{f} }}{{\partial \tilde{\mu }_{\lambda } }}} \right)}\nolimits_{\alpha }^{ - } = \inf \left\{ {\bigcup\limits_{\alpha \in (0,1]} \alpha \left[ { - \frac{{\theta :[\tilde{\Theta }_{R} \cdot (m_{N} \tilde{R}_{aN} + m_{W} \tilde{R}_{aW} )]}}{{\sqrt {2\pi } \tilde{\sigma }_{Z} }}\exp \left[ { - \frac{1}{2}\left( {\frac{{\tilde{\mu }_{Z} }}{{\tilde{\sigma }_{Z} }}} \right)^{2} } \right]} \right]_{\alpha } } \right\}$$
  34 $$\mathop {\left( {\frac{{\partial \tilde{P}_{f} }}{{\partial \tilde{\mu }_{\lambda } }}} \right)}\nolimits_{\alpha }^{ + } = \sup \left\{ {\bigcup\limits_{\alpha \in (0,1]} \alpha \left[ { - \frac{{\theta :[\tilde{\Theta }_{R} \cdot (m_{N} \tilde{R}_{aN} + m_{W} \tilde{R}_{aW} )]}}{{\sqrt {2\pi } \tilde{\sigma }_{Z} }}\exp \left[ { - \frac{1}{2}\left( {\frac{{\tilde{\mu }_{Z} }}{{\tilde{\sigma }_{Z} }}} \right)^{2} } \right]} \right]_{\alpha } } \right\}$$
  35 $$\mathop {\left( {\frac{{\partial \tilde{P}_{f} }}{{\partial \tilde{\sigma }_{\lambda } }}} \right)}\nolimits_{\alpha }^{ - } = \inf \left\{ {\bigcup\limits_{\alpha \in (0,1]} \alpha \left[ { - \frac{{\theta :[\tilde{\Theta }_{R} \cdot (m_{N} \tilde{R}_{aN} + m_{W} \tilde{R}_{aW} )]^{2} \tilde{\mu }_{Z} \tilde{\sigma }_{T} }}{{\sqrt {2\pi } \tilde{\sigma }_{Z}^{3} }}\exp \left[ { - \frac{1}{2}\left( {\frac{{\tilde{\mu }_{Z} }}{{\tilde{\sigma }_{Z} }}} \right)^{2} } \right]} \right]_{\alpha } } \right\}$$
  36 $$\mathop {\left( {\frac{{\partial \tilde{P}_{f} }}{{\partial \tilde{\sigma }_{\lambda } }}} \right)}\nolimits_{\alpha }^{ + } = \sup \left\{ {\bigcup\limits_{\alpha \in (0,1]} \alpha \left[ { - \frac{{\theta :[\tilde{\Theta }_{R} \cdot (m_{N} \tilde{R}_{aN} + m_{W} \tilde{R}_{aW} )]^{2} \tilde{\mu }_{Z} \tilde{\sigma }_{T} }}{{\sqrt {2\pi } \tilde{\sigma }_{Z}^{3} }}\exp \left[ { - \frac{1}{2}\left( {\frac{{\tilde{\mu }_{Z} }}{{\tilde{\sigma }_{Z} }}} \right)^{2} } \right]} \right]_{\alpha } } \right\}$$
4. Fuzzy random sensitivity model for calculating the uncertainty coefficient of the model.
  37 $$\mathop {\left( {\frac{{\partial \tilde{P}_{f} }}{{\partial \tilde{\mu }_{{\Theta_{R} }} }}} \right)}\nolimits_{\alpha }^{ - } = \inf \left\{ {\bigcup\limits_{\alpha \in (0,1]} \alpha \left[ { - \frac{{\theta :(m_{N} \tilde{\lambda }_{N} \tilde{R}_{aN} + m_{W} \tilde{\lambda }_{W} \tilde{R}_{aW} + \tilde{\mu }_{N} R_{gN} + \tilde{\mu }_{W} R_{gW} )}}{{\sqrt {2\pi } \tilde{\sigma }_{Z} }}\exp \left[ { - \frac{1}{2}\left( {\frac{{\tilde{\mu }_{Z} }}{{\tilde{\sigma }_{Z} }}} \right)^{2} } \right]} \right]_{\alpha } } \right\}$$
  38 $$\mathop {\left( {\frac{{\partial \tilde{P}_{f} }}{{\partial \tilde{\mu }_{{\Theta_{R} }} }}} \right)}\nolimits_{\alpha }^{ + } = \sup \left\{ {\bigcup\limits_{\alpha \in (0,1]} \alpha \left[ { - \frac{{\theta :(m_{N} \tilde{\lambda }_{N} \tilde{R}_{aN} + m_{W} \tilde{\lambda }_{W} \tilde{R}_{aW} + \tilde{\mu }_{N} R_{gN} + \tilde{\mu }_{W} R_{gW} )}}{{\sqrt {2\pi } \tilde{\sigma }_{Z} }}\exp \left[ { - \frac{1}{2}\left( {\frac{{\tilde{\mu }_{Z} }}{{\tilde{\sigma }_{Z} }}} \right)^{2} } \right]} \right]_{\alpha } } \right\}$$
  39 $$\mathop {\left( {\frac{{\partial \tilde{P}_{f} }}{{\partial \tilde{\sigma }_{{\Theta_{R} }} }}} \right)}\nolimits_{\alpha }^{ - } = \inf \left\{ {\bigcup\limits_{\alpha \in (0,1]} \alpha \left[ { - \frac{{\theta :(m_{N} \tilde{\lambda }_{N} \tilde{R}_{aN} + m_{W} \tilde{\lambda }_{W} \tilde{R}_{aW} + \tilde{\mu }_{N} R_{gN} + \tilde{\mu }_{W} R_{gW} )^{2} \tilde{\mu }_{Z} \tilde{\sigma }_{\omega } }}{{\sqrt {2\pi } \tilde{\sigma }_{Z}^{3} }}\exp \left[ { - \frac{1}{2}\left( {\frac{{\tilde{\mu }_{Z} }}{{\tilde{\sigma }_{Z} }}} \right)^{2} } \right]} \right]_{\alpha } } \right\}$$
  40 $$\mathop {\left( {\frac{{\partial \tilde{P}_{f} }}{{\partial \tilde{\sigma }_{{\Theta_{R} }} }}} 
\right)}\nolimits_{\alpha }^{ + } = \sup \left\{ {\bigcup\limits_{\alpha \in (0,1]} \alpha \left[ { - \frac{{\theta :(m_{N} \tilde{\lambda }_{N} \tilde{R}_{aN} + m_{W} \tilde{\lambda }_{W} \tilde{R}_{aW} + \tilde{\mu }_{N} R_{gN} + \tilde{\mu }_{W} R_{gW} )^{2} \tilde{\mu }_{Z} \tilde{\sigma }_{\omega } }}{{\sqrt {2\pi } \tilde{\sigma }_{Z}^{3} }}\exp \left[ { - \frac{1}{2}\left( {\frac{{\tilde{\mu }_{Z} }}{{\tilde{\sigma }_{Z} }}} \right)^{2} } \right]} \right]_{\alpha } } \right\}$$
5. Fuzzy random sensitivity model for the soil layer depth.
  41 $$\mathop {\left( {\frac{{\partial \tilde{P}_{f} }}{{\partial \tilde{\mu }_{H} }}} \right)}\nolimits_{\alpha }^{ - } = \inf \left\{ {\bigcup\limits_{\alpha \in (0,1]} \alpha \left[ {\frac{13}{{1000\sqrt {2\pi } \tilde{\sigma }_{Z} }}\exp \left[ { - \frac{1}{2}\left( {\frac{{\tilde{\mu }_{Z} }}{{\tilde{\sigma }_{Z} }}} \right)^{2} } \right]} \right]_{\alpha } } \right\}$$
  42 $$\mathop {\left( {\frac{{\partial \tilde{P}_{f} }}{{\partial \tilde{\mu }_{H} }}} \right)}\nolimits_{\alpha }^{ + } = \sup \left\{ {\bigcup\limits_{\alpha \in (0,1]} \alpha \left[ {\frac{13}{{1000\sqrt {2\pi } \tilde{\sigma }_{Z} }}\exp \left[ { - \frac{1}{2}\left( {\frac{{\tilde{\mu }_{Z} }}{{\tilde{\sigma }_{Z} }}} \right)^{2} } \right]} \right]_{\alpha } } \right\}$$
  43 $$\mathop {\left( {\frac{{\partial \tilde{P}_{f} }}{{\partial \tilde{\sigma }_{H} }}} \right)}\nolimits_{\alpha }^{ - } = \inf \left\{ {\bigcup\limits_{\alpha \in (0,1]} \alpha \left[ {\frac{{169\tilde{\mu }_{Z} \tilde{\sigma }_{{\mu_{g} }} }}{{1000000\sqrt {2\pi } \tilde{\sigma }_{Z}^{3} }}\exp \left[ { - \frac{1}{2}\left( {\frac{{\tilde{\mu }_{Z} }}{{\tilde{\sigma }_{Z} }}} \right)^{2} } \right]} \right]_{\alpha } } \right\}$$
  44 $$\mathop {\left( {\frac{{\partial \tilde{P}_{f} }}{{\partial \tilde{\sigma }_{{\mu_{g} }} }}} \right)}\nolimits_{\alpha }^{ + } = \sup \left\{ {\bigcup\limits_{\alpha \in (0,1]} \alpha \left[ {\frac{{169\tilde{\mu }_{Z} \tilde{\sigma }_{{\mu_{g} }} }}{{1000000\sqrt {2\pi } \tilde{\sigma }_{Z}^{3} }}\exp \left[ { - \frac{1}{2}\left( {\frac{{\tilde{\mu }_{Z} }}{{\tilde{\sigma }_{Z} }}} \right)^{2} } \right]} \right]_{\alpha } } \right\}$$
  Among them:
  45 $$\tilde{\mu }_{Z} = \mu_{{\tilde{\Theta }_{R} }} \cdot (m_{N} \mu_{{\tilde{\lambda }_{N} }} \mu_{{\tilde{R}_{aN} }} + m_{W} \mu_{{\tilde{\lambda }_{W} }} \mu_{{\tilde{R}_{aW} }} + \mu_{{\tilde{\mu }_{N} }} R_{gN} + \mu_{{\tilde{\mu }_{W} }} R_{gW} ) - 0.013\mu_{{\tilde{H}}}$$
  46 $$\tilde{\sigma }_{Z} = \sqrt {\begin{array}{*{20}l} {(\left. {\frac{{\partial \tilde{Z}}}{{\partial \tilde{\Theta }_{R} }}} \right|_{{\tilde{\mu }_{{\Theta_{R} }} }} \cdot \tilde{\sigma }_{{\Theta_{R} }} )^{2} + (\left. {\frac{{\partial \tilde{Z}}}{{\partial \tilde{\lambda }_{N} }}} \right|_{{\tilde{\mu }_{{\lambda_{N} }} }} \cdot \tilde{\sigma }_{{\lambda_{N} }} )^{2} + (\left. {\frac{{\partial \tilde{Z}}}{{\partial \tilde{R}_{{{\text{a}}N}} }}} \right|_{{\tilde{\mu }_{{R_{aN} }} }} \cdot \tilde{\sigma }_{{R_{aN} }} )^{2} + (\left. {\frac{{\partial \tilde{Z}}}{{\partial \tilde{\lambda }_{W} }}} \right|_{{\tilde{\mu }_{{\lambda_{W} }} }} \cdot \tilde{\sigma }_{{\lambda_{W} }} )^{2} } \hfill \\ { + (\left. {\frac{{\partial \tilde{Z}}}{{\partial \tilde{R}_{{{\text{a}}W}} }}} \right|_{{\tilde{\mu }_{{R_{aW} }} }} \cdot \tilde{\sigma }_{{R_{aW} }} )^{2} + (\left. {\frac{{\partial \tilde{Z}}}{{\partial \tilde{\mu }_{N} }}} \right|_{{\tilde{\mu }_{{\mu_{{_{N} }} }} }} \cdot \tilde{\sigma }_{{\mu_{{_{N} }} }} )^{2} + (\left. {\frac{{\partial \tilde{Z}}}{{\partial \tilde{\mu }_{W} }}} \right|_{{\tilde{\mu }_{{\mu_{{_{W} }} }} }} \cdot \tilde{\sigma }_{{\mu_{{_{W} }} }} )^{2} + (\left. {\frac{{\partial \tilde{Z}}}{{\partial \tilde{H}}}} \right|_{{\tilde{\mu }_{H} }} \cdot \tilde{\sigma }_{H} )^{2} } \hfill \\ \end{array} }$$

## Results and discussion

### Engineering examples

**Figure 2:**
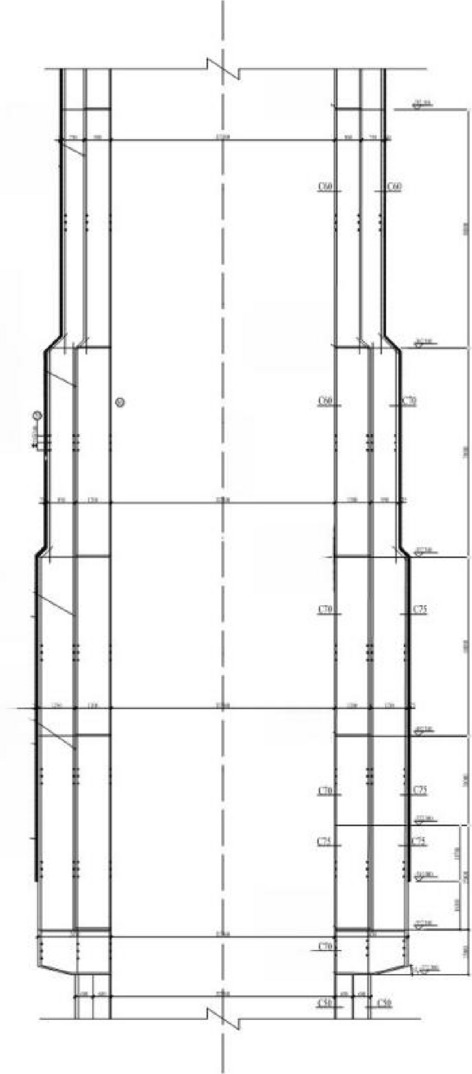
Shaft structure of the main well.

A large mine in Lianghuai mining area adopts the freezing method for construction and shaft development, through deep alluvium. The main shaft, secondary shaft, and wind shaft all adopt a reinforced concrete double wall structure. The structure of the main shaft are shown in Fig. 2. The frozen soil and shaft structure parameters during construction are shown in Tables 2 and 3. Among them, the uncertainty distribution characteristics of fuzzy random parameters are shown in Table 4.

**Table 2:** Frozen geotechnical parameter.

Mine	Depth (m)	Lithology	Water content (%)	Freezing wellbore thickness (m)	Mean temperature(°C)
Main shaft	425	Calcareous clay	24.21	11.6	− 18.9
	480	Calcareous clay	25.66	11.4	− 18.7
	523	Clay	26.02	11.5	− 18.6
Auxiliary shaft	406	Calcareous clay	25.31	11.2	− 19.1
	452	Clay	27.32	10.8	− 19.6
	507	Clay	26.82	11.4	− 19.8
Air shaft	421	Calcareous clay	24.58	11.8	20.6
	466	Calcareous clay	25.19	12.1	21.1
	530	Clay	26.73	11.7	21.3

**Table 3:** Structural parameters of outer wellbore.

Mine	Depth (m)	Grade of concrete	Inner radius (mm)	Outer radius (mm)	Inner reinforcement ratio(%)	Outer reinforcement ratio(%)
Main shaft	391–430	C60	5250	6300	0.528	0.481
	430–490	C70	5250	6550	0.562	0.538
	490–545	C75	5250	6550	0.604	0.573
Auxiliary shaft	400–420	C65	5000	6000	0.682	0.667
	420–480	C65	5200	6250	0.722	0.709
	480–544	C70	5200	6250	0.750	0.696
Air shaft	400–430	C65	5050	6450	0.652	0.684
	430–490	C65	5050	6450	0.738	0.705
	490–538	C70	5100	6500	0.756	0.732

**Table 4:** The uncertainty characteristics of parameters.

Uncertainty characteristic distribution	Uncertainty of compressive strength of concrete axis	Uncertainty of circumferential reinforcement ratio	Uncertainty of thickness to diameter ratio	Uncertainty of calculating coefficient	Uncertainty of soil layer depth
Mean	0.992	1.041	1.033	0.850	0.956
Standard deviation	0.185	0.074	0.254	0.217	0.032
Coefficient of variation	0.129	0.072	0.245	0.254	0.178

The cumulative freezing period was assumed to be 185 days. The coefficient for enhancing the strength of cement in the inner and outer strata was *m*_*N*_ = 1.5, and *m*_*W*_ = 1.8. The yield strengths of the inner and outer wall steel bars were *R*_*gN*_ = 335 N/mm^2^, and *R*_*gW*_ = 400 N/mm^2^.

The fuzzy random limit state values were $\widetilde{b}={\bigcup }_{\alpha \in (\text{0,1}]}\alpha [0.65+(\alpha -1)0.1,\hspace{0.33em}0.65+(1-\alpha )0.1]$. The constraint level was set to 0.75.

**Figure 3:**
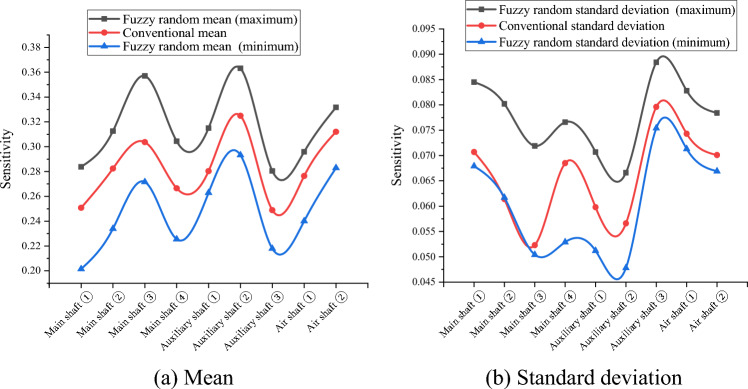
Fuzzy random sensitivity radar map of concrete axial compressive strength.

**Figure 4:**
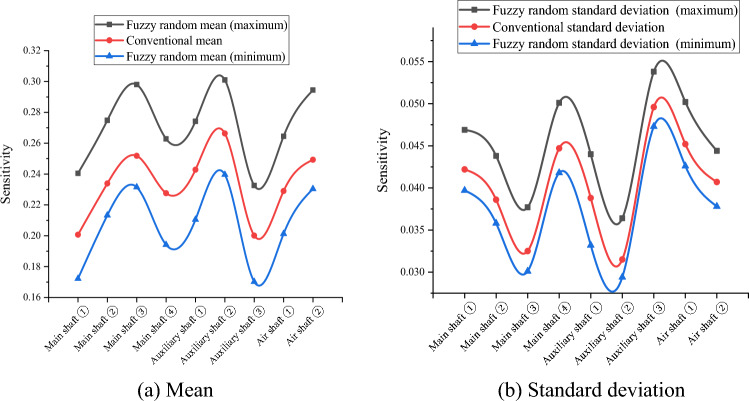
Fuzzy random sensitivity radar map of wellbore thickness to diameter ratio.

**Figure 5:**
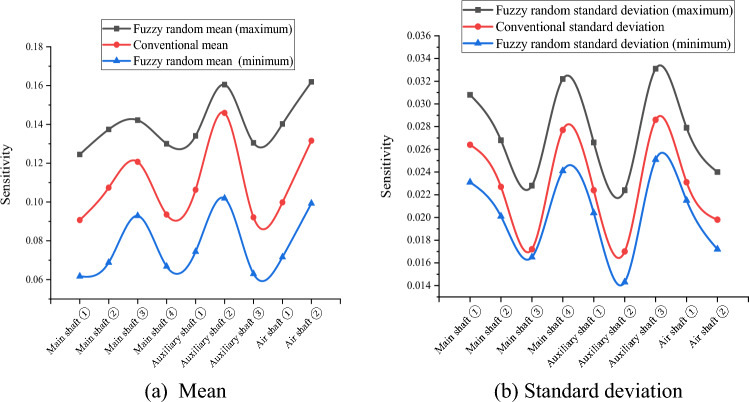
Fuzzy random sensitivity radar map of shaft circumferential reinforcement ratio.

**Figure 6:**
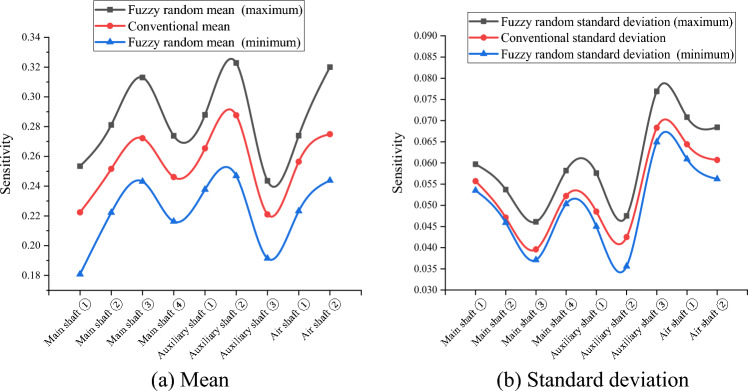
Fuzzy random sensitivity radar map of calculation model uncertainty coefficient.

**Figure 7:**
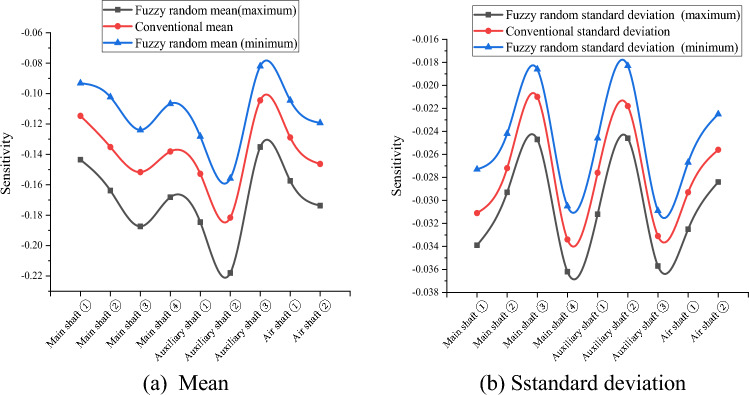
Fuzzy random sensitivity radar map of soil depth.

Because the variables in $\widetilde{H},{\widetilde{R}}_{aN},{\widetilde{R}}_{aW},{\widetilde{\mu }}_{N},{\widetilde{\mu }}_{W},{\widetilde{\lambda }}_{N}, and {\widetilde{\lambda }}_{W}$ are uncertain in practical engineering, their fuzzy randomization generations were included in the fuzzy stochastic reliability sensitivity model established in this study. MATLAB software (MathWorks MATLAB R2023b, https://ww2.mathworks.cn/products/matlab.html) was used to compile the HMM and EM algorithm for big data to calculate the fuzzy random reliability sensitivity of the main well, auxiliary well, and wind well structures in each buried depth section of the deep alluvial layer^31–34^. After 1000 iterations, the Monte Carlo method calculations and comparison results of the conventional sensitivity and fuzzy random sensitivity of each parameter are shown in Tables 5, 6, 7, 8 and 9 and Figs. 3, 4, 5, 6 and 7.

**Table 5:** Fuzzy random sensitivity of axial compressive strength of concrete.

Mine	Deep/m	Conventional mean sensitivity	Conventional standard deviation sensitivity	Fuzzy random mean sensitivity		Fuzzy random standard deviation sensitivity	
				$\mathop {\left( {\frac{{\partial \tilde{P}_{f} }}{{\partial \tilde{\mu }_{{x_{i} }} }}} \right)}\nolimits_{\alpha }^{ - }$	$\mathop {\left( {\frac{{\partial \tilde{P}_{f} }}{{\partial \tilde{\mu }_{{x_{i} }} }}} \right)}\nolimits_{\alpha }^{ + }$	$\mathop {\left( {\frac{{\partial \tilde{P}_{f} }}{{\partial \tilde{\sigma }_{{x_{i} }} }}} \right)}\nolimits_{\alpha }^{ - }$	$\mathop {\left( {\frac{{\partial \tilde{P}_{f} }}{{\partial \tilde{\sigma }_{{x_{i} }} }}} \right)}\nolimits_{\alpha }^{ + }$
Main shaft	391–430	0.2508	0.0707	0.2015	0.2838	0.0679	0.0845
	430–490	0.2824	0.0614	0.2340	0.3126	0.0617	0.0802
	490–545	0.3037	0.0523	0.2716	0.3570	0.0504	0.0719
	400–420	0.2665	0.0685	0.2255	0.3044	0.0529	0.0766
Auxiliary shaft	420–480	0.2803	0.0598	0.2629	0.3150	0.0512	0.0707
	480–544	0.3249	0.0566	0.2932	0.3631	0.0478	0.0666
	400–430	0.2489	0.0796	0.2179	0.2805	0.0754	0.0884
Air shaft	430–490	0.2764	0.0743	0.2401	0.2959	0.0713	0.0828
	490–538	0.3120	0.0701	0.2829	0.3317	0.0669	0.0784

**Table 6:** Fuzzy random sensitivity of wellbore thickness-to-diameter ratio.

Mine	Deep/m	Conventional mean sensitivity	Conventional standard deviation sensitivity	Fuzzy random mean sensitivity		Fuzzy random standard deviation sensitivity	
				$\mathop {\left( {\frac{{\partial \tilde{P}_{f} }}{{\partial \tilde{\mu }_{{x_{i} }} }}} \right)}\nolimits_{\alpha }^{ - }$	$\mathop {\left( {\frac{{\partial \tilde{P}_{f} }}{{\partial \tilde{\mu }_{{x_{i} }} }}} \right)}\nolimits_{\alpha }^{ + }$	$\mathop {\left( {\frac{{\partial \tilde{P}_{f} }}{{\partial \tilde{\sigma }_{{x_{i} }} }}} \right)}\nolimits_{\alpha }^{ - }$	$\mathop {\left( {\frac{{\partial \tilde{P}_{f} }}{{\partial \tilde{\sigma }_{{x_{i} }} }}} \right)}\nolimits_{\alpha }^{ + }$
Main shaft	391–430	0.2007	0.0422	0.1722	0.2405	0.0397	0.0469
	430–490	0.2339	0.0386	0.2133	0.2748	0.0358	0.0438
	490–545	0.2518	0.0325	0.2315	0.2980	0.0301	0.0377
	400–420	0.2276	0.0447	0.1942	0.2628	0.0418	0.0501
Auxiliary shaft	420–480	0.2428	0.0388	0.2105	0.2742	0.0332	0.044
	480–544	0.2663	0.0315	0.2396	0.3010	0.0294	0.0364
	400–430	0.2001	0.0496	0.1702	0.2326	0.0473	0.0538
Air shaft	430–490	0.2290	0.0452	0.2013	0.2645	0.0426	0.0502
	490–538	0.2493	0.0407	0.2304	0.2944	0.0378	0.0444

**Table 7:** Fuzzy random sensitivity of shaft circumferential reinforcement ratio.

Mine	Deep/m	Conventional mean sensitivity	Conventional standard deviation sensitivity	Fuzzy random mean sensitivity		Fuzzy random standard deviation sensitivity	
				$\mathop {\left( {\frac{{\partial \tilde{P}_{f} }}{{\partial \tilde{\mu }_{{x_{i} }} }}} \right)}\nolimits_{\alpha }^{ - }$	$\mathop {\left( {\frac{{\partial \tilde{P}_{f} }}{{\partial \tilde{\mu }_{{x_{i} }} }}} \right)}\nolimits_{\alpha }^{ + }$	$\mathop {\left( {\frac{{\partial \tilde{P}_{f} }}{{\partial \tilde{\sigma }_{{x_{i} }} }}} \right)}\nolimits_{\alpha }^{ - }$	$\mathop {\left( {\frac{{\partial \tilde{P}_{f} }}{{\partial \tilde{\sigma }_{{x_{i} }} }}} \right)}\nolimits_{\alpha }^{ + }$
Main shaft	391–430	0.0907	0.0264	0.0617	0.1245	0.0231	0.0308
	430–490	0.1074	0.0227	0.0688	0.1374	0.0201	0.0268
	490–545	0.1207	0.0172	0.0929	0.1422	0.0165	0.0228
	400–420	0.0935	0.0277	0.0668	0.1300	0.0241	0.0322
Auxiliary shaft	420–480	0.1063	0.0224	0.0744	0.1341	0.0204	0.0266
	480–544	0.1459	0.0170	0.1019	0.1605	0.0143	0.0224
	400–430	0.0921	0.0286	0.0630	0.1305	0.0251	0.0331
Air shaft	430–490	0.0998	0.0231	0.0716	0.1402	0.0215	0.0279
	490–538	0.1316	0.0198	0.0993	0.1619	0.0172	0.0240

**Table 8:** Fuzzy random sensitivity of calculation model uncertainty coefficient.

Mine	Deep/m	Conventional mean sensitivity	Conventional standard deviation sensitivity	Fuzzy random mean sensitivity		Fuzzy random standard deviation sensitivity	
				$\mathop {\left( {\frac{{\partial \tilde{P}_{f} }}{{\partial \tilde{\mu }_{{x_{i} }} }}} \right)}\nolimits_{\alpha }^{ - }$	$\mathop {\left( {\frac{{\partial \tilde{P}_{f} }}{{\partial \tilde{\mu }_{{x_{i} }} }}} \right)}\nolimits_{\alpha }^{ + }$	$\mathop {\left( {\frac{{\partial \tilde{P}_{f} }}{{\partial \tilde{\sigma }_{{x_{i} }} }}} \right)}\nolimits_{\alpha }^{ - }$	$\mathop {\left( {\frac{{\partial \tilde{P}_{f} }}{{\partial \tilde{\sigma }_{{x_{i} }} }}} \right)}\nolimits_{\alpha }^{ + }$
Main shaft	391–430	0.2224	0.0557	0.1808	0.2534	0.0535	0.0597
	430–490	0.2516	0.0471	0.2222	0.2811	0.0459	0.0537
	490–545	0.2722	0.0396	0.2431	0.3130	0.0371	0.0461
	400–420	0.2461	0.0522	0.2163	0.2738	0.0503	0.0582
Auxiliary shaft	420–480	0.2654	0.0485	0.2377	0.2879	0.045	0.0576
	480–544	0.2876	0.0425	0.2469	0.3228	0.0356	0.0475
	400–430	0.2210	0.0683	0.1914	0.2436	0.0649	0.0769
Air shaft	430–490	0.2564	0.0644	0.2232	0.2739	0.0609	0.0708
	490–538	0.2749	0.0607	0.2438	0.3200	0.0562	0.0684

**Table 9:** Fuzzy random sensitivity of soil depth.

Mine	Deep/m	Conventional mean sensitivity	Conventional standard deviation sensitivity	Fuzzy random mean sensitivity		Fuzzy random standard deviation sensitivity	
				$\mathop {\left( {\frac{{\partial \tilde{P}_{f} }}{{\partial \tilde{\mu }_{{x_{i} }} }}} \right)}\nolimits_{\alpha }^{ - }$	$\mathop {\left( {\frac{{\partial \tilde{P}_{f} }}{{\partial \tilde{\mu }_{{x_{i} }} }}} \right)}\nolimits_{\alpha }^{ + }$	$\mathop {\left( {\frac{{\partial \tilde{P}_{f} }}{{\partial \tilde{\sigma }_{{x_{i} }} }}} \right)}\nolimits_{\alpha }^{ - }$	$\mathop {\left( {\frac{{\partial \tilde{P}_{f} }}{{\partial \tilde{\sigma }_{{x_{i} }} }}} \right)}\nolimits_{\alpha }^{ + }$
Main shaft	391–430	− 0.1147	− 0.0311	− 0.0932	− 0.1435	− 0.0273	− 0.0339
	430–490	− 0.1352	− 0.0272	− 0.1023	− 0.1638	− 0.0242	− 0.0293
	490–545	− 0.1517	− 0.021	− 0.124	− 0.1874	− 0.0186	− 0.0247
	400–420	− 0.1381	− 0.0334	− 0.1067	− 0.1682	− 0.0305	− 0.0362
Auxiliary shaft	420–480	− 0.1528	− 0.0276	− 0.1282	− 0.1846	− 0.0246	− 0.0312
	480–544	− 0.1816	− 0.0218	− 0.1558	− 0.218	− 0.0183	− 0.0246
	400–430	− 0.1045	− 0.0331	− 0.082	− 0.1352	− 0.0309	− 0.0357
Air shaft	430–490	− 0.1289	− 0.0293	− 0.1045	− 0.1574	− 0.0267	− 0.0325
	490–538	− 0.1463	− 0.0256	− 0.1193	− 0.1737	− 0.0225	− 0.0284

According to Tables 5, 6, 7, 8 and 9, the fuzzy random sensitivity analysis of each parameter of the frozen wellbore fully considers the gradual evolution of underground engineering from stability to instability on the basis of the fuzzy random reliability theory, thus improving the previous cognition that there are only two isolated states of structural stability. By numerical calculation of the HMM and EM algorithm, it was found that the traditional sensitivity values of each parameter are included in the fuzzy random sensitivity interval^35,36^. For example, the conventional mean sensitivities of the axial compressive strength of the main shaft concrete were 0.2508, 0.2824, 0.3037, and 0.2665, and the fuzzy random mean sensitivity ranges were [0.2015, 0.2838], [0.2340, 0.3126], [0.2716, 0.3570], and [0.2255, 0.3044]. The conventional standard deviation sensitivities of the compressive strength of the main shaft concrete were 0.0707, 0.0614, 0.0523, and 0.0685, and the fuzzy random standard deviation sensitivity ranges were [0.0679, 0.0845], [0.0617, 0.0802], and [0.0504, 0.0719]. [0.0529, 0.0766]. The above analysis shows that the fuzzy random sensitivity of the overall structural reliability of the reinforced concrete double-layer wellbore optimized by the algorithm represents the fuzzy random degree of influence of the main parameters on the overall structural reliability of the wellbore in the form of a fuzzy interval value. This is consistent with the conventional sensitivity value and is more reasonable from an engineering perspective than the traditional fixed value representation method of sensitivity.

**Figure 8:**
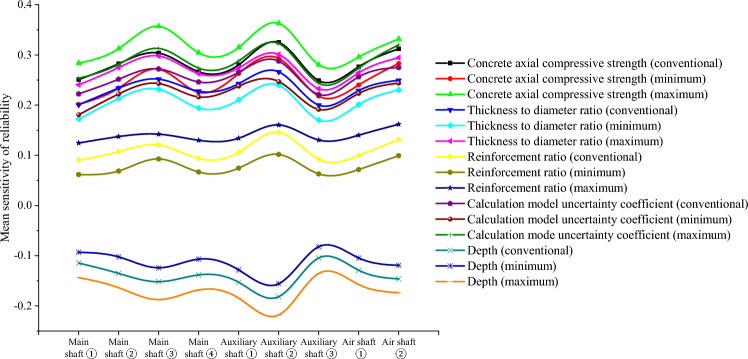
Mean comparison of fuzzy random sensitivity.

**Figure 9:**
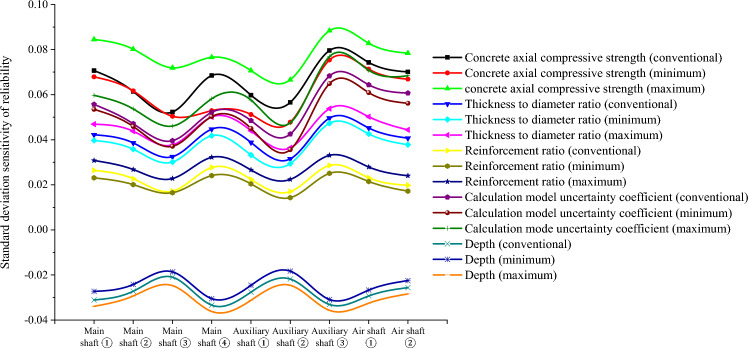
Standard deviation comparison of fuzzy random sensitivity.

To further analyze the fuzzy random sensitivity and distinguish the importance of each parameter to the overall reliability of the wellbore structure^37,38^, the conventional sensitivity mean value and standard deviation of each major parameter can be compared with the fuzzy random sensitivity mean value and fuzzy random standard deviation^39,40^. The results are shown in Figs. 8 and 9.

**Figure 10:**
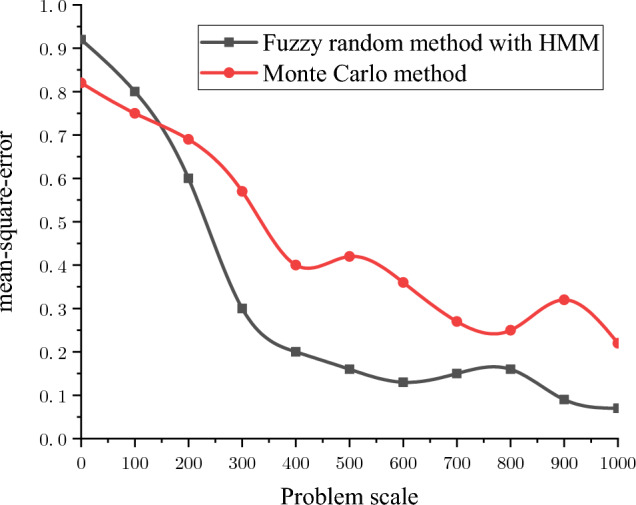
Efficiency comparison .

Through a comprehensive comparison of the fuzzy random sensitivity of each parameter, it can be seen that the main factors affecting the reliability of the entire structure of the frozen shaft of reinforced concrete, in order of importance, are the compressive strength of concrete, uncertainty coefficient of the calculation model, thickness-to-diameter ratio, depth of the soil layer, and reinforcement ratio. In addition, the fuzzy random sensitivity analysis in this study had positive and negative symbolic characteristics. The positive/negative sign indicates that the parameter change had a positive/negative relationship with the reliability of the overall wellbore structure. In future frozen shaft excavation practices, more attention must be paid to the main factors affecting the reliability of the shaft and distinguishing the direction characteristics of the influence. For example, the fuzzy random sensitivity numerical calculation results for the reliability of a reinforced concrete shaft in a deep alluvial layer show that the concrete compressive strength, uncertainty coefficient of the calculation model, thickness-to-diameter ratio, and reinforcement ratio are positively correlated, whereas the soil depth is negatively correlated with the overall shaft reliability. In addition, in order to further verify the robustness of the optimization algorithm, the convergence efficiency of the fuzzy random method with HMM and the traditional Monte Carlo method is compared through the above engineering cases, and the results are shown in Fig. 10. It can be seen that the fuzzy random method is more robust and efficient than the traditional method (Supplementary information).

## Conclusions

1. To address the shortcomings of traditional reliability in characterizing the stability of deep underground structures, the AFSOM method was improved to analyze fuzzy random reliability, which is more consistent with the actual working conditions. To reflect the influence of uncertain parameters on the safety and reliability of the entire structure, the conventional sensitivity analysis model was optimized using fuzzy random reliability theory, and the mean and standard deviation calculation models of the fuzzy random reliability sensitivity were established.
2. Based on the fuzzy random analysis of the overall structural resistance and load of the frozen wellbore in the deep alluvial layer, the uncertainties of different variable parameters were comprehensively considered, and the numerical characteristics of the fuzzy random variables were replaced by the HMM and EM algorithm of big data. The fuzzy random sensitivity optimization model of the concrete compressive strength, thick-diameter ratio, reinforcement ratio, uncertainty coefficient of the calculation model, and soil depth on the reliability of the entire wellbore structure were obtained.
3. Engineering examples show that the optimized fuzzy random sensitivity expresses the degree of influence of each parameter on the overall reliability of the wellbore structure with a fuzzy interval value, which is consistent with the conventional sensitivity value but also provides more engineering rationality than the traditional fixed value form of sensitivity. Therefore, this method can provide a more effective reference for the safe construction of deep underground structures.
4. By comparing the numerical calculation results of the fuzzy random sensitivity of the main parameters, it can be seen that the main factors affecting the reliability of the overall structure of the deep alluvial reinforced concrete double-layer wellbore are, in order of importance, the concrete compressive strength, uncertainty coefficient of the calculation model, thickness-to-diameter ratio, soil depth, and reinforcement ratio. Simultaneously, the fuzzy random sensitivity analysis also has the characteristics of positive and negative symbols, indicating that the concrete compressive strength, uncertainty coefficient of the calculation model, thickness-to-diameter ratio, and reinforcement rate have a positive relationship with wellbore reliability, whereas the soil depth has a negative relationship with wellbore reliability.

### Supplementary Information

Supplementary Information.

## Data Availability

All data, models, and code generated or used during the study appear in the submitted article.

## Abbreviations

AFOSM: Advanced first order second moment
EM: Expectation-maximization
HMM: Hidden Markov model
PDF: Probability density function
