# Supplementary material for: Fuzzy random sensitivity analysis for the overall structure reliability of reinforced concrete freezing wellbores in deep alluvium based on hidden Markov model
Source: Sci Rep. 2024 Jul 6;14:15584. doi: 10.1038/s41598-024-65914-4 (PMC11227586; doi:10.1038/s41598-024-65914-4)
Supplement: Supplementary file 1 — Supplementary Information. [file 41598_2024_65914_MOESM1_ESM.docx]

Supplementary Appendix

1. Traditional reliability theory

Generally, the reliability of a building refers to the reliability of its structure. Structural reliability refers to the probability that the engineering structure will complete its predetermined function over a specified period and under specific conditions. This is a probability measure of the reliability of the engineering structure.

According to the load-resistance model of the reliability theory, the functions of a structure are defined as [11, 12]:

$Z=R(x_{1}, x_{2},\ldots,x_{m})-S(y_{1}, y_{2},\ldots, y_{n})$ (1)

In Eq. (1), *Z* is the structural function, *R* is the resistance of the structure (i.e., its bearing capacity), *S* is the load borne by the structure, $x_{1}, x_{2},\ldots,x_{m}$ are the parameters affecting the resistance, and $y_{1}, y_{2},\ldots, y_{n}$ are the parameters affecting the load.

Structural functions are classified according to their performance state and typically have three working states. The relationships between the states in a rectangular coordinate system are illustrated in Fig. 1.


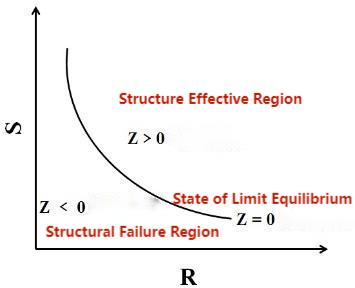


Fig.1 State diagram of performance function.

In Fig. 1, the *Z* > 0 region indicates that the resistance is greater than the load. It is called the effective zone of the structure. The zone *Z* < 0 indicates that the resistance is less than the load, which is the failure zone of the structure. The curve represents the limit state where the load and the resistance are equal, and the limit state equation can be expressed by the function *Z* = 0. By establishing the limit state equation, the limit state design of the structure can be conducted, and the corresponding reliability index of the structure can be obtained.

If both the resistance *R* and load *S* are subject to a normal distribution or can be transformed into a normal distribution, then the function *Z* = *R* - *S* is also subject to a normal distribution. Let the mean of *R* and *S* be $\mu_{R} and \mu_{S}$, respectively, and the standard deviation be $\sigma_{R} and \sigma_{S}$, respectively. According to the nature of the numerical characteristics of random variables, the mean and standard deviation of the structural function *Z* are:

$\mu_{Z}=\mu_{R}-\mu_{S}$ (2)

$\sigma_{Z}=\sqrt{{\sigma_{R}}^{2}+{\sigma_{S}}^{2}}$ (3)

Therefore, the structural failure probability can be expressed as

$P_{f}=P(Z<0)=\int_{-\infty}^{0} \frac{1}{\sqrt{2\pi}\sigma_{Z}}\exp[-\frac{(z-\mu_{z})^{2}}{2{\sigma_{z}}^{2}}]dz$ (4)

According to the standard normal distribution transformation of the above equation, the failure probability, $P_{f}$, can be obtained as follows:

$P_{f}=P(Z<0)=\int_{-\infty}^{-\frac{\mu_{z}}{\sigma_{z}}} \frac{1}{\sqrt{2\pi}}\exp[-\frac{t^{2}}{2}]dt=\Phi(-\beta)$ (5)

In Eq. (5), *β* is the reliability index of the structure, and $\Phi$ is the standard normal distribution function.

By substituting Eqs. (2) and (3) into Eq. (5), based on the properties of the normal distribution function, the reliability index and reliability expression can be obtained as follows:

$\beta=\frac{\mu_{z}}{\sigma_{z}}=\frac{\mu_{R}-\mu_{S}}{\sqrt{{\sigma_{R}}^{2}+{\sigma_{S}}^{2}}}$ (6)

$P_{S}=1-P_{f}=\Phi(\beta)$ (7)

In Eq. (7), *P_S_* is the probability of structural reliability, which is referred to as the reliability.

2. Reliability fuzzy random optimization

Many studies have found that the transition from an effective state to an invalid state is not instantaneous but gradual. Therefore, the traditional reliability equation of state *Z* can be fuzzy randomized [13].

$\tilde{Z}=g(\tilde{x}_{1},\tilde{x}_{2},\cdots,\tilde{x}_{m})=\tilde{R}-\tilde{S}=\tilde{b}$ (8)

In Eq. (8), $\tilde{R} and \tilde{S}$ are the resistance and load, respectively, of the entire fuzzy randomization structure, $\tilde{b}$ is a very small fuzzy random threshold, and $\tilde{x_{1}},\tilde{x_{2}},\ldots,\tilde{x_{m}}$ is each fuzzy random parameter in the structure.

To align to the actual situation in the project as closely as possible, bounded closed fuzzy random numbers near zero are typically used to characterize the fuzzy random threshold $\tilde{b}$. The membership function of this fuzzy number *X* can be expressed as [14]:

$\mu_{\alpha}(\tilde{x})=\left\{ \begin{aligned} &\frac{1}{d}(x+d) x\in[-d ， 0] \\ &-\frac{1}{d}(x-d) x\in[0 ，d] \\ & \\ &0 other \end{aligned} \right.$ (9)

In Eq. (9),$\alpha\in(0,1]$ is the fuzzy random constraint level of the threshold value $\tilde{b}$; *d* is the range of the corresponding horizontal cut set region. Therefore, after fuzzy randomization, Eq. (1) becomes

$\tilde{Z}_{\alpha}=\tilde{R}_{\alpha}-\tilde{S}_{\alpha}=\tilde{b}_{\alpha}$ (10)

In Eq. (10), the right subscript *α* is the corresponding constraint level, and Eq. (10) is transformed according to the fuzzy interval operation as follows:

$\left\{ \begin{aligned} &{Z_{\alpha}}^{-}={R_{\alpha}}^{-}-{S_{\alpha}}^{+}={b_{\alpha}}^{-} \\ &{Z_{\alpha}}^{+}={R_{\alpha}}^{+}-{S_{\alpha}}^{-}={b_{\alpha}}^{+} \end{aligned} \right.$ (11)

In Eq. (11), the right superscripts - and + are the corresponding left and right extreme values of each variable, respectively.

According to the definition of reliability and the decomposition theorem of fuzzy sets, the structural fuzzy random reliability index $\tilde{\beta}$ can be expressed as follows:

$\tilde{\beta}=\frac{\mu_{R}-\mu_{S}}{\sqrt{{\sigma_{R}}^{2}+{\sigma_{S}}^{2}}}=\bigcup_{\alpha\in(0,1]} \alpha[{\beta_{\alpha}}^{-},{\beta_{\alpha}}^{+}]$ (12)

In Eq. (12), $\mu_{R}and \mu_{S}$ are the mean values of $\tilde{R}$ and $\tilde{S}$, respectively; $\sigma_{R} and \sigma_{S}$ are the standard deviations, respectively; and $\cup$ is the interval of the cut set at its constrained level. Similarly, the fuzzy random failure probability $\tilde{P_{f}}$ and fuzzy random reliability $\tilde{P_{S}}$ are:

$\tilde{P}_{f}=\bigcup_{\alpha\in(0,1]} \alpha[{{P_{f}}_{\alpha}}^{-},{{P_{f}}_{\alpha}}^{+}]$ (13)

$\tilde{P}_{s}=1-\bigcup_{\alpha\in(0,1]} \alpha[{{P_{f}}_{\alpha}}^{-},{{P_{f}}_{\alpha}}^{+}]$ (14)

It can be seen that the constraint level and fuzzy random threshold are introduced into the traditional equation of state, and the traditional reliability algorithm is fuzzy randomized to obtain the corresponding solution interval equation.

$\tilde{\Theta}_{R}$ uncertainty coefficient of the calculation model

$\tilde{R}_{aN}, \tilde{R}_{aW}$ compressive strength of the inner and outer shaft

$\tilde{\mu}_{N} , \tilde{\mu}_{W}$ratio of the annular reinforcements of the inner and outer shaft

$\tilde{\lambda}_{N} ,\tilde{\lambda}_{W}$ thickness-to-diameter ratio of the inner and outer shaft

$m_{N} , m_{W}$ the inner and outer wall concrete strength enhancement coefficients

$R_{gN} ,R_{gW}$ the strengths of the steel bars in the inner and outer shaft

$inf(\cdot)$ minimum value of fuzzy random function

$\sup(\cdot)$ maximum value of fuzzy random function

**Nomenclature**

*Z* the structural function

*R* the resistance of the structure

*S* the load borne by the structure

$P_{f}$ the structural failure probability

$\Phi$ the standard normal distribution function

$\beta$ the reliability index

$P_{S}$ the probability of reliability

$\tilde{X}$ fuzzy random variable

$X^{-}$ left extreme values

$X^{+}$ right extreme values

$\cup$ the interval of the cut set at its constrained level

*b* a very small fuzzy random threshold

$\alpha$ the corresponding constraint level

$\theta$: parameter expectation estimation

$\tilde{R_{k}}$ fuzzy random value of the ultimate bearing capacity
